# Irregularities of the Teeth

**Published:** 1885-02

**Authors:** Norman W. Kingsley


ARTICLE II.

### IRREGULARITIES OF THE TEETH.

BY DR. NORMAN KINGSLEY.

Read before the Odonto-Chirurgical Society of Scotland.

With advancing civilization there is an apparent increase of deformities of the dental arch.

In the higher social scale it is exceptional to find a young person with a perfectly developed and regular row of teeth, set in a well-formed and rounded arch. More commonly, departures from this type will be found of every grade, more or less pronounced, exhibiting some of the phases of narrowed jaws, or teeth protruding, over-lapping, or crowded in every conceivable mal-arrangement. In many instances the cause has some direct connection with other evils that seem inseparable from a state of high civilization.

A close observer for a generation will have seen a multitude of cases which had no apparent local cause, were not of an inherited origin, and could only be attributable to constitutional conditions developing in the individual.

As a general statement, the finer the nervous organization, the more precocious or brilliant the intellect, the greater will be the tendency to mental deformity. The converse is true of feeble-minded people, who, having a fair physique, will show well-rounded jaws and regular dental arches. The exceptions to the latter statement are found among those cases of hopeless idiocy where the whole organization, as well as the intellect, is depraved.

Many peculiarities are of an inherited origin, so far as the individual is concerned; but what caused the initial departure from a normal type in preceeding generations would be impossible to determine. More readily, perhaps, than any other deformities of the human organization are dental irregularities transmissible, and departures from a normal type in the parents re-appear in the children in an aggravated form.

Irregularities that require treatment never appear in the deciduous teeth. The deciduous dental arch is always well-formed and symmetrical. It is only in the second set that deformities make their appearance, and it is exceptional that such peculiarities can be foreseen and prevented. It cannot be determined with any certainty before eruption that a dental arch is going to be abnormal, the causes being generally hidden and remote.

The normal type of the dental arch describes a regular line. The arch may be wider or narrower, varrying somewhat in individuals or races, but the line will be an easy, graceful curve, without break or tendency to form an angle. Within certain limits, a narrow dental arch, as associated with certain features, may become the perfection of beauty; while, with another form of head and face, the widest development may be equally pleasing.

That which is recognized now as the standard or full measure of beauty, as well as utility, is not unlike that which existed in the remotest historic ages, nor different from that which is now exhibited among all communities not degenerated by luxury or vice.

Abnormalties include such a shape of the arch as is not in harmony with the surrounding features; all crowding and twisting of the teeth; and all departures from a regular line in their positions. One form of irregularity seems to be due to unwise or premature extraction of the deciduous canines of the upper jaw. In the ordinary course of nature, these teeth should be the last to drop out. If extracted long anterior to their period of shedding, the permanent bicuspids are liable to encroach upon the domain of the canines, and thus deprive them of their place in the arch. Such a mal-position can be foreseen and prevented.

Another abnormalty of the superior dental arch which can be prevented, is the result of thumb-sucking, or its equivalent, in the earlier years of childhood. The effect of such a habit is to protrude all the teeth in the front part of the mouth. This deformity will not show itself until the eruption of the permanent teeth, sometimes even after the practice which caused it may have ceased. In one instance which came under the author's observation, a mother of good social position had nursed from her breast a daughter until she was nine years old, the result being that the six upper front teeth were protruding so that the lips could not be closed. But a large proportion of dental irregularities cannot be predetermined with certainty even where there is an hereditary tendency, and can be corrected only when they develop.

An observer with limited experience may often be misled by the appearance of teeth as they first erupt. They may seem to be growing out of the line of the arch, and that a permanent irregularity is inevitable; but many such cases need no interference. If left to themselves, they will approach regularity, and often assume their true position, unless the occlusion of the antagonizing teeth prevents them. But interferance is demanded as soon after eruption as it becomes certain that a deformity is inevitable. There is then no longer justification for delay; for after that period every year increases the difficulties, both pathological and mechanical, and prejudices the stability of the dental apparatus.

But all irregularities in the position of the teeth are not deformities which demand treatment; there are many departures from the normal type where neither the utility nor the beauty of these organs, nor the symmetry of surrouning features, is seriously affected by such mal-position.

The regulation of teeth involves often the wearing of fixtures which cannot be removed and cleansed as frequently as the health of the mouth demands. Their continued presence may provoke caries of the teeth, and a prolonged treatment may seriously impair a nervous system; therefore regulating teeth should not be undertaken without due consideration. Such a consideration must recognize the age sex, and family type, and the relation of the features; the cause of displacement, the constitution of the teeth, and the ravages of decay; the efficiency of the mastication apparatus, the enunciation of the voice, the risk of inflammation, and the destruction of pulps; the systemic condition and endurance of the patient; the time required, and the annoyance to which he would be subjected; and the means and appliances for correction; and finally, the character and permanency of the changes wrought.

Regulating teeth may be undertaken under favorable circumstances at any age short of maturity; but, all things considered, the most desirable period to begin the correction of an extended irregularity is when the cuspidate and second molars are fully erupted. The occlusion of the teeth is an important factor in determining the permanency of the change. All attempts at correction at any age will be folly, unless the antagonizing teeth upon occlusion will serve to hold them in their new positions.

Success in treatment is based upon the fact that the teeth are placed upon the maxillæ, surrounded by vascular, eclastic, bony processes, which are easily moved or absorbed under pressure, and that reproduction of bone will follow such conditions, and make the teeth solid in their new locations. The possibilities under favorable conditions, within certain limits, are almost unbounded. Narrow jaws may be widened, protruding jaws made to recede, individual teeth moved considerable distances, and teeth elongated or shortened, or twisted in their sockets. The success of skillful efforts in this direction has been triumphant.

Some of the most marked cases have been where the face has been deformed by a protruding or receding jaw, either upper or lower. Strictly speaking, when this occurs with the upper jaw, it is not the maxilla which is at fault, but rather the whole dental arch. Such a condition in the lower jaw is more likely to arise from a mal-articulation at the joint; but in either case, when taken at the proper age, they are quite emenable to treatment.

It is possible, in correcting deformities or irregularities of the dental arch, to create a deformity of the external features. It is not always advisable to attempt to alter the form and expression of a mouth where the condition is an inherited peculiarity—being a part of the family type—and where the change would involve prolonged effort, possible breaking up of a good articulation of masticating organs, and with the knowledge that nature will be constantly making an effort to return to the inherited type. In hereditary cases of extensive character which have been delayed until at or near maturity, we can never feel certain but that the original tendency to mal-position, so long unbroken, will re-assert itself at any time we abandon retaining fixtures.

Upon general principles, it is desirable to retain every sound tooth in the mouth, yet there are many cases of crowded dentition where the removal of a tooth upon each side of the jaw is justifiable. The retention of every tooth in the mouth is not necessary to the efficiency of the masticating apparatus, is not required to maintain the contour of the jaws, and the loss of certain teeth produces no visible external effect. The articulation of masticating organs is of more importance than their number, and a limited number of grinding teeth fitting closely upon occlusion will be of greater benefit to the individual than a mouthful of teeth with the articulation disturbed.

In the abnormality arising from the mixing of inharmonious types, as the small jaw of one parent with the large teeth of the other—resulting in a crowded condition—it is very doubtful if the arch can be made regular without the extraction of some of the teeth. It requires a judgment founded upon long experience to decide always upon the wisdom of extraction, and when such conviction is reached, one may be equally at a loss as to the choice of the teeth to be removed. It is a disputed point which of the teeth behind the canines can be best spared from the mouth. There are so many considerations to be taken into account that it is hardly possible to lay down any rule of universal application. If the sixth year molars are badly decayed, their removal would be indicated. If they were sound, and also the bicuspids, there might be no greater reason for their removal than either of the bicuspids. In fact, sound molars in the jaw are of more value as masticating organs than equally sound bicuspids.

As a rule, extraction of any teeth from a pinched or V-shaped jaw before it is widened, would be likely to prove bad practice. Certainly, the extraction of any teeth from the sides of the jaw in such cases for the purpose of correcting or improving the condition, without immediate subsequent steps being instituted to widen the arch, would be most unscientific and detrimental. Cases are not uncommon which show that a pair of any of the teeth in the mouth may be removed to correct an irregularity, excepting the canines of both jaws, and the superior central incisors. It would be an inconceivable case which would justify the extraction of the superior central incisors; but the upper lateral incisors, and any pair of the lower incisors, may be removed in certain cases without serious detriment to the appearance of the mouth.

The most frequent exhibition of irregularity in the lower jaws is confined to the incisors and canines. The canines are too prominent, and the incisors are crowded and lapping. Most of such cases cannot be corrected by enlarging the arch; the overlapping of the superior incisors forbids. The remedy is comparatively simple. The extraction of one of the centrals is innicated. This will give the required room, and enable the canines to be drawn in; and at the same time the space made by extraction is closed. No matter what the mal-arrangement of the incisors may be, it is almost always better to extract one of the centrals to correct it. When the irregularity is corrected, the extracted tooth will never be missed.

The treatment of irregularities is almost entirely mechanical. To the anatomical, physiological, and pathological knowledge required of the operator, there must be added a knowledge of mechanism and an ingenuity to apply it. Levers, pulleys, inclined planes, wedges and elastics, singly and in combination, are required for this purpose. It is quite impossible for one to overcome a complicated case of irregularity who has not a comprehension of each and all of these instrumentalities.

So far as pressure itself is concerned, it is immaterial from whence is is derived. The same weight, force, or power will produce the same result. It is only a matter of convenience what source shall be employed. For widening a narrowed arch, a jack screw is the most effective means, and can be used to spread one tooth only, or all the teeth on both sides, according as it is applied. Wedges driven between the teeth will enlarge the arch. Levers with elastics are used to twist teeth in their sockets, and an inclined plane can be made to move teeth laterally.

The application of such apparatus to the movement of teeth is one of the most responsible duties the dentist is called upon to perform. Each and every one of these mechanical powers can be made to do his bidding, and equally each one of these may become a formidable engine of disaster. When applied in the mouth, they should have constant watchfulness and care. Not one of them but, in the hands of empires, would cause the destruction of those valuable organs they can be made to conserve.—*London Dental Record.*

